# The Inorganic Component as a Possible Marker for Quality and for Authentication of the Hazelnut’s Origin

**DOI:** 10.3390/ijerph17020447

**Published:** 2020-01-09

**Authors:** Paolo Inaudi, Agnese Giacomino, Mery Malandrino, Carmela La Gioia, Eleonora Conca, Tanmoy Karak, Ornella Abollino

**Affiliations:** 1 Department of Drug Science and Technology, University of Torino, 10125 Torino, Italy; paolo.inaudi718@edu.unito.it (P.I.); ornella.abollino@unito.it (O.A.); 2 Department of Chemistry, University of Torino, 10125 Torino, Italy; mery.malandrino@unito.it (M.M.); carmela.lagioia@unito.it (C.L.G.); eleonora.conca@unito.it (E.C.); 3 Upper Assam Advisory Centre, Tea Research Association, Dikom 786101, Dibrugarh, Assam, India; t.karak@tocklai.net

**Keywords:** hazelnuts, metals, authentication, technological impact, marker, chemometric treatments

## Abstract

The inorganic component of hazelnuts was considered as a possible marker for geographical allocation and for the assessment of technological impact on their quality. The analyzed samples were Italian hazelnuts of the cultivar *Tonda Gentile Romana* and Turkish hazelnuts of the cultivars *Tombul*, *Palaz* and *Çakildak*. The hazelnuts were subjected to different drying procedures and different conservative methods. The concentration of 13 elements, namely Ba, Ca, Cu, Fe, K, Mg, Mn, Na, Ni, P, Sn, Sr and Zn, were quantified by inductively coupled plasma optical emission spectroscopy (ICP-OES). All the samples were previously digested in a microwave oven. Before proceeding with the analysis of the samples, the whole procedure was optimized and tested on a certified reference material. The results show that the inorganic component: (i) can represent a fingerprint, able to identify the geographical origin of hazelnuts, becoming an important quality marker for consumer protection; (ii) is strongly influenced by the treatments undergone by the investigated product during all the processing stages. A pilot study was also carried out on hazelnuts of the cultivar *Tonda Gentile Trilobata Piemontese*, directly harvested from the plant during early development to maturity and analyzed to monitor the element concentration over time.

## 1. Introduction

The hazelnut is a fruit widely used in the confectionery industry. It is grown worldwide, but primarily in Turkey and Italy; Turkey produces 67% of the total, while Italy ranks second with 13% [1]. The remaining 20% of the production takes place in countries such as the USA, Spain, Azerbaijan, Georgia, and Iran. The United States produces about 90% of their total national amount in the Willamette Valley (Oregon), where they exclusively grow the *Barcelona* and *Ennis* varieties. In Spain, hazelnuts are grown in Catalonia, and the most widespread cultivar is *Negret*, which is mainly used by local industries.

Hazelnut cultivation occupies an important position in Italian agriculture, as Italy produces different quality cultivars such as the *Tonda Gentile Trilobata* from Piedmont that gained the Protected Geographical Indication (PGI) label under the name *Nocciola Piemonte* in 1996 from the European Union (EU) [2]. The cultivar *Nocciola Romana* was awarded the Protected Designation of Origin (PDO) mark in 2009, which refers in particular to the dried fruit, in shell or shelled forms, belonging to the species *Corylus avellana*, variety *Tonda Gentile Romana* and *Nocchione*, grown in the provinces of Rome and Viterbo.

Nevertheless, in recent years, the production of hazelnuts has also quickly increased in other countries due to large investments for improving agricultural techniques and the selection of new cultivars. This situation has led to a competition between Italian hazelnuts and others, since the former are more expensive, due to their appreciated sensory properties.

Most (90%) of the hazelnuts produced in the world are used in the confectionery industry, so it is important to find a marker for quality authentication [3].

All aspects of the hazelnut have been studied for years; in particular, important studies were carried out in order to find a better method for shelling the fruits [4,5], to minimize the loss of material, for geographical characterization [6,7], and to evaluate the influence of fertilizers [8,9]. Besides, monitoring the concentrations of aflatoxins, highly toxic secondary metabolites produced by fungi present in hazelnuts [10], and analyzing the fatty acid composition [11] is essential. Significant works have been done to evaluate the volatile components present in the aroma of hazelnuts and the influence of roasting on the quality of the hazelnuts [12,13,14], while others were focused on authentication studies on the basis of the content of lanthanides [15].

Hazelnuts were completely ignored in some studies of trace element level [16,17] surveys. In the last two decades, the situation has gradually improved, with several reports focusing on essential and trace elements in nuts and seeds becoming available [18,19].

This may reflect the increasing recognition of nuts and seeds as important sources of some essential elements (e.g., B, Cu, Fe, Mn, Se and Zn) in human nutrition, especially for vegetarians [19,20], as well as the growing popularity of various healthy diets (including the Mediterranean diet) that rely on frequent use of these constituents.

Macroelement and microelement contents have been studied in hazelnuts [19,21] and similar studies have been carried out in pine nuts [22,23], almonds [24] and nuts [9,25,26,27,28].

The aim of this work is to monitor the inorganic components in hazelnuts in order to: (i) evaluate the possibility of using metal composition as a marker of product origin, and ii) to assess the impact that different treatments (drying, storing, roasting) could have on hazelnut composition.

Different techniques are commonly used to assess hazelnut metal composition (i.e., atomic absorption spectroscopy (AAS) [27,28,29], inductively coupled plasma mass spectrometry (ICP-MS) [30,31] or ICP optical emission spectroscopy (ICP-OES) [19]). Other studies have performed the analysis of hazelnuts for authentication purposes, using, for example, simple sequence repeats (SSR) [32,33], near infrared spectroscopy (NIR) [34], two-dimensional gas chromatography fast scanning quadrupole mass spectrometry [13] (GC–GC-QMS) or liquid chromatography [35] (LC-QqQ-MS/MS), in which organic substances were determined, and ICP sector field mass spectrometry (ICP-SFMS) [28] and ^1^H-NMR [36].

In this study, attention was focused on the elements detectable using ICP-OES, namely Ba, Ca, Cu, Fe, K, Mg, Mn, Na, Ni, P, Sn, Sr and Zn, to identify if their concentration is sufficient to determine the origin of hazelnuts. Other techniques, such as ICP-MS, permit us to determine macro- and microelements, rare earth elements, and isotopes, but require longer time, expert personnel and/or higher cost with respect to ICP-OES.

Hazelnuts belonging to different cultivars, namely Italian hazelnuts from the Lazio region (cultivar: *Tonda Gentile Romana*) and Turkish hazelnuts from the Ordu region (cultivars: *Tombul*, *Palaz* and *Çakildak*) were considered in this work. The choice of these two types of samples was dictated by market needs: Italian confectionery companies have to supplement local hazelnut production by purchasing foreign hazelnuts, especially Turkish ones, to satisfy national needs. In these two countries, different drying procedures were adopted. Samples were provided in order to assess whether the preservation processes had the same impact on hazelnuts belonging to different cultivars and dried in different ways. This aspect is very important to guarantee the quality of the final product independently of the raw material used.

Moreover, some samples were roasted in a laboratory to determine the effect of this process on the inorganic component. Roasting is a crucial step in hazelnut processing, since it has positive effects for food safety (e.g., reduction of allergenicity and aflatoxins, water activity [37,38], improved flavor and color of kernels, and it leads to a desirable crunchy texture [39]). On the contrary, the roasting process affects the composition of the raw fruits. Several papers have been published previously about this aspect, but they are focused on sugars, organic acids, condensed tannins, free phenolic acids and fatty acid profiles [40,41]. No study about the impact of this step on the hazelnut elemental composition is available.

This study was made possible thanks to the access granted by a local farmer: Italian hazelnuts from the Piedmont region (cultivar: *Tonda Gentile Trilobata Piemontese*) were sampled directly from the plant at different times during the period of ripening of the fruit to study the evolution of the inorganic elements over time. Moreover, fruits harvested both in the middle of the plantation (Pm) and on the roadside (Pr) were compared to see if road traffic is able to influence the concentration of anthropogenic elements typically emitted by road traffic. The study on the Piedmont hazelnut samples was added here to give a complete picture of all the aspects related to the content of metals and other trace elements throughout the processing chain.

A chemometric analysis of the experimental results was performed by principal component analysis (PCA).

## 2. Materials and Methods

Sample dissolution was performed in polytetrafluoroethylene (PTFE) bombs, with a Milestone MLS-1200 Mega (Milestone, Sorisole, Italy) microwave laboratory unit.

Sample analyses were carried out with a Perkin Elmer Optima 7000 (Perkin Elmer, Norwalk, CT, USA) inductively coupled plasma optical emission spectrometer (ICP-OES).

Analytical grade reagents were used throughout the experiments. Standard metal solutions were prepared from concentrated Merck Titrisol stock solutions (Merck, Darmstadt, Germany).

High purity water (HPW) obtained from a Milli-Q apparatus (Millipore, Burlington, MA, USA) was used for the preparation of samples as well as standard solutions.

### 2.1. Samples

#### 2.1.1. Reference Material

Unfortunately, no certified reference materials of nuts are available on the market. In order to check the accuracy of the experimental procedure and to assess the effect of vegetal matrices on element determination, the Standard Reference Material (SRM) 1573a of the National Institute of Standards and Technology (NIST), namely Tomato Leaves, was analyzed. The analyzed elements were Al (598 ± 12 mg·kg^−1^), As (0.112 ± 0.004 mg·kg^−1^), B (33.3 ± 0.7 mg·kg^−1^), Ba (63 mg·kg^−1^), Ca (5.05 ± 0.09%), Cd (1.52 ± 0.04 mg·kg^−1^), Co (0.57 ± 0.02 mg·kg^−1^), Cr (1.99 ± 0.06 mg·kg^−1^), Cu (0.57 ± 0.02 mg·kg^−1^), Fe (368 ± 7 mg·kg^−1^), Hg (0.034 ± 0.004 mg·kg^−1^), K (2.70 ± 0.05%), Mg (1.2%), Mn (246 ± 8 mg·kg^−1^), Na (136 ± 4 mg·kg^−1^), Ni (1.59 ± 0.07 mg·kg^−1^), P (0.216 ± 0.004%), Sb (0.063 ± 0.006 mg·kg^−1^), Se (0.054 ± 0.003 mg·kg^−1^), Sr (85 mg·kg^−1^), V (0.835 ± 0.010 mg·kg^−1^), and Zn (30.9 ± 0.7 mg·kg^−1^).

#### 2.1.2. Main Study

Hazelnuts from Turkey, in particular from the Ordu region, belonging to the cultivars *Tombul*, *Palaz* and *Çakildak* (designated as O-samples in this work), and from Italy, in particular from the Lazio region, belonging to the cultivar *Tonda Gentile Romana* (designated as R-samples in this work), were considered for the main study of this work. All the samples were provided by a confectionery company. All samples had the same caliber, that is an average diameter of 13–15 mm.

The samples were subjected to a drying procedure. The hazelnuts from the Ordu region were purchased from Turkey, so they were dried following the local tradition, i.e., the fruits were sun-dried in the field immediately after the harvest, typically at 30–35 °C in the local summer climate, for about three weeks. The hazelnuts from Italy were dried by the producer in artificial driers at different temperatures: one batch (R-D1) was dried at 35–40 °C (to simulate the drying conditions applied to the Turkish hazelnut), while a second batch (R-D2) was dried at 15–20 °C following the procedure commonly adopted by the Italian farmer.

By the time the hazelnuts arrive at the confectionery company, they have experienced five different types of storage methods in which atmospheric conditions and temperature were varied: (i) 3–8 °C/humidity: 60–70% (A-Method); (ii) 15–20 °C/humidity: 60–70% (B-Method); (iii) 3–8 °C/humidity: air (C-Method); (iv) 15–20 °C /vacuum (D-Method); (v) temperatures below zero (E-Method).

The samples were analyzed as received from the farmer (T0) and after one, two and four months in each type of storage from the company (T1, T2 and T4). The mass of the hazelnuts does not change during the storage period.

The whole list of considered samples is reported in Table 1.

#### 2.1.3. Evaluation of the Roasting Step

The roasting conditions generally used for hazelnuts range from 100 °C to 160 °C for 10 min to 60 min [42,43]. In this study, some R and O samples were roasted for 15 min at 105 °C to evaluate the effect of this process on the quality of the fruit (Table 2).

All the hazelnuts were chopped before the digestion and analyzed in duplicate.

#### 2.1.4. Pilot Study on the Trend of the Inorganic Content from Early Development to Maturity

A local company (located in the Piedmont region, Italy) allowed us to sample hazelnuts in their fields. In this case, the fruit belongs to the cultivar *Tonda Gentile Trilobata* (designated as P-samples in this study). The hazelnuts were collected monthly during the ripening of the fruit (from June to September 2017). These samples were collected separately from trees situated in the middle of the plantation (Pm) and on the roadside (Pr).

P-samples, with the exception of the hazelnuts collected in the first month which had no shell, were shelled immediately after harvesting. All samples were dried in a laboratory stove for one hour at 60 °C, chopped, and then subjected to the same digestion and analysis procedures of the other samples.

### 2.2. Procedures

For the digestion, aliquots of 0.5 g of each sample were treated with 5 mL of a reaction mixture composed of HNO_3_ and H_2_O_2_ (4:1), according to the literature [27].

Initially, the SRM sample was used to optimize the whole procedure. Regarding the digestion step, the addition of a known amount of HPW (1 mL or 3 mL) to each aliquot of SRM in the vessel before adding the digestion mixture [44] was tested. A known concentration of lutetium (Lu) was also added to the SRM samples as an internal standard to correct the concentration obtained.

After the heating program (2 min at 250 W; 2 min at 0 W, 6 min at 250 W, 5 min at 400 W, 5 min at 600 W) and ventilation step (25 min), the resulting solutions were filtered on Whatman Grade 5 cellulose filters and transferred into 50 mL Falcon tubes, where HPW was added to a final volume of 30 mL.

The solutions were analyzed by ICP-OES. The calibration was performed with standard solutions prepared in aliquots of sample blanks diluted at the same ratios as the sample solutions. Standard solutions were periodically analyzed and their signals were used to correct the drift of instrumental sensitivity. The limits of detection (LoD) were estimated as three times the standard deviation of the blank.

### 2.3. Chemometric Treatments

Principal component analysis (PCA) was carried out with the aid of the XLSTAT4.4 software package (Addinsoft, Paris, France), a Microsoft Excel plug-in. Unscrambler X 10.2 (Camo Analytics, Oslo, Norway) was employed for auto-scaling the dataset and for substituting values below LoDs with estimated values.

## 3. Results

### 3.1. Choice of Experimental Conditions

In order to optimize the procedures used in this study, 0.5 g of SRM 1573a were digested with and without wetting the sample with 1 mL or 3 mL of HPW before the addition of the HNO_3_/H_2_O_2_ mixture. Moreover, the possible correction of the final element concentration with the recovery of the internal standard (Lu) was also tested. Table 3 shows the recoveries obtained in the different experiments.

It is possible to observe that the best results were obtained by wetting the sample with 3 mL of HPW; no improvement was observed by applying the internal standard correction. Therefore, each sample was wet with 3 mL of HPW before the digestion and the addition of Lu was not performed.

### 3.2. Main Study

Table S1 (Supplementary Material) reports the concentrations of Ba, Ca, Cu, Fe, K, Mg, Mn, Na, Ni, P, Sn, Sr and Zn in the analyzed samples. All of the concentrations are related to the dry weight. The concentration of Co and Cr was below the LoD (0.08 mg·kg^−1^ and 0.03 mg·kg^−1^, respectively) in all the samples. Firstly, the metal content measured for the O- and R-samples at T0, i.e., after the drying step and before the storage step, was compared. A significant difference (by ANOVA) between the two cultivars is observed: Turkish hazelnuts have a higher content of Ba and P, while *Romana* samples have higher concentrations of Fe, K, Mn, Na, Sn and Sr. The only element for which the concentrations are not significantly different is Cu.

By comparing R-samples after the two different drying procedures, it is possible to observe that R-D2-samples show a higher concentration of Mn and, to a lesser extent, of Fe, Sn and Zn than R-D1, while R-D1 presents a higher content of all the other elements examined.

By calculating the total amount of elements in the samples, it can also be noted that *Romana* hazelnuts contain a higher metal content than Turkish ones. Applying an ANOVA test, no significant differences were observed between the R-samples dried by the different procedures.

Tables S2–S6 and Tables S7–S16 report the composition of O- and R-samples at different storage times for each type of storage method. The metal content measured at the corresponding T0 is also reported to better observe possible variations during the storage time. A good storage procedure should maintain the characteristics of the fresh hazelnuts, so no variation in term of macro- and micronutrients is expected.

**Figure 1:**
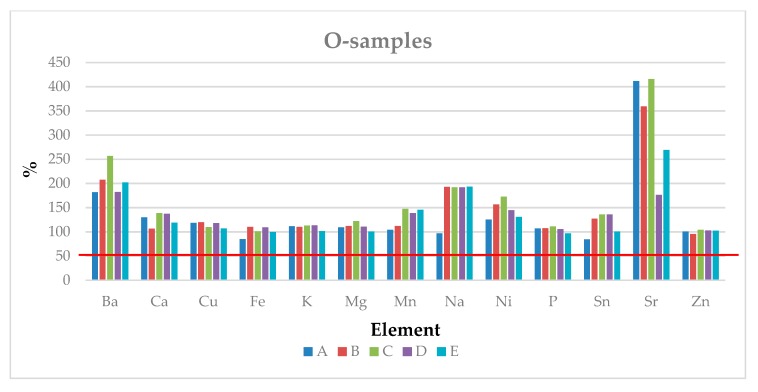
Concentration (%) of each element in the O-samples after four months of storage following the different methods (A–E), expressed with respect to T0 (100%).

Figure 1 shows the results obtained for O-samples at the storage time T4, expressed as a percentage in comparison to the concentration found at T0 (horizontal red line: 100%) in order to compare the effect of each storage method.

Regarding O-samples, it is possible to see an increase of the metal content over the time for all the storage methods (with the exception of Fe, Na and Sn for A-Method (3–8 °C/humidity: 60–70%) and Zn for B-Method (15–20 °C/humidity: 60–70%). In the case of Ba, Ca, Cu, Mn, Ni, Sn and Sr, it is possible to observe a marked increase with respect to the initial composition. The explanation for this behavior is not obvious: a possible contamination of the samples from the packaging adopted during the storage time may have occurred. Among these elements, Cu, Mn and Ni are well known for their catalytic effect on the oxidation process, which can cause an oxidative rancidity of the hazelnuts.

After applying an ANOVA test on the concentration found for O-T4 samples after the different storage methods, only the concentrations of K, Sn and Zn were not significantly different in comparison to O-T0, independently of the storage method adopted. The content of other elements varies randomly depending on the conservative method used.

**Figure 2:**
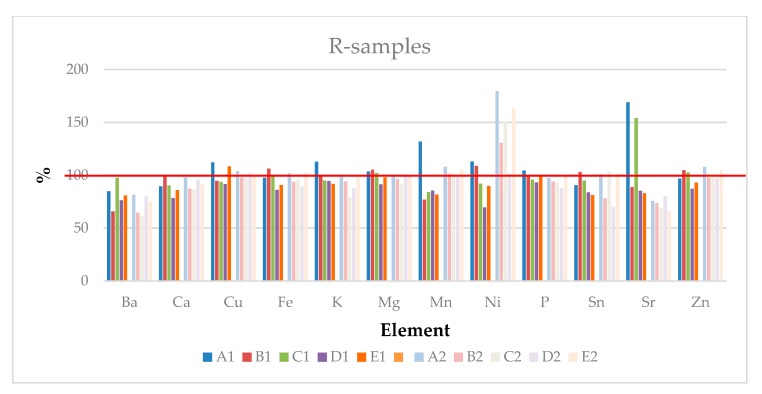
Concentration (%) of each element in the R-samples after four months of storage following the different methods (A–E), expressed with respect to T0 (100%).

Regarding the R-samples, Figure 2 shows the concentrations in the T4-specimens, reported as a percentage in comparison with the corresponding concentration in T0-samples.

The total metal content remains quite constant or seems to decrease over the time.

In regards to the R-D1-samples, after applying an ANOVA: (i) the concentrations of K, Mg, Na, P and Sn T4 samples were not significantly different from T0 for all the conservative methods; (ii) the B-Method (15–20 °C/humidity: 60–70%) seems to be the best in order to preserve the inorganic composition during storage; and (iii) the D-Method appears to be the worst storage procedure, as it causes the greatest variation in the element concentrations.

By applying an ANOVA on R-D2-T4 samples, it is possible to see that the concentration of all elements, with the exception of Ca and Ni, were not significantly different independent of the conservative method adopted. This behavior could be due to a better stabilization of the hazelnut dried at a lower temperature compared to the other samples. On the contrary, it is possible to observe that Ni, a catalyst of the rancidity processes, increases with all methods, except the D-Method (15–20 °C/vacuum).

The A-Method (3–8 °C/humidity: 60–70%) seems to be the best, since the concentration of 12 elements in the T4 samples are not significantly different in comparison to D2-T0. It is not possible to define which is the worse storage procedure, since each element has a characteristic trend.

In any case, at the quantitative level, it is difficult to determine which drying or storage procedure should be taken as the optimum.

**Figure 3:**
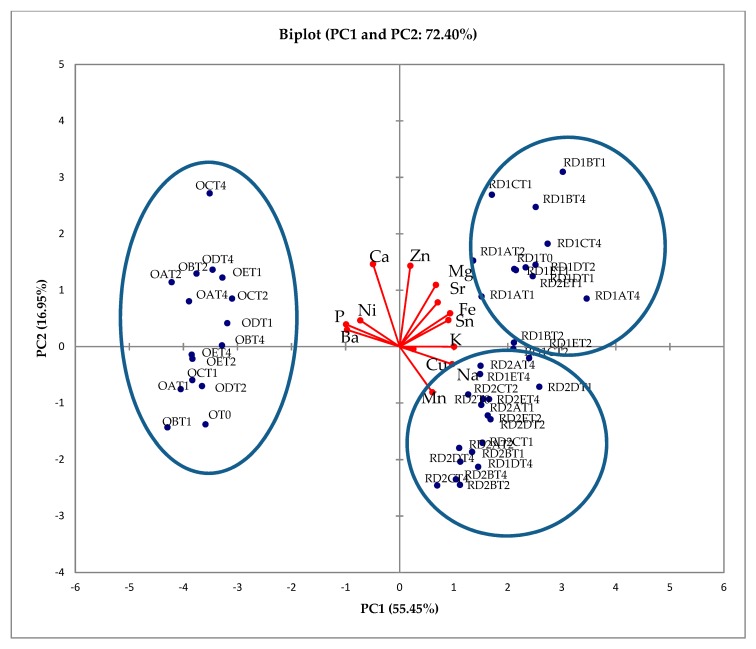
Biplot obtained by principle component analysis (PCA) (PC1 vs. PC2) on the whole dataset.

The experimental data obtained in this study were subjected to principal component analysis (PCA). The biplot obtained by PCA is shown in Figure 3 (PC1 vs. PC2). The first and the second PCs retain 55.45% and 16.95% of the total variance, respectively.

It is possible to observe a good separation of Italian and Turkish hazelnuts according to their different inorganic composition: O-samples contain the highest levels of Ba, Ni and P, while R-samples are characterized by high concentrations of Fe, K, Mg, Mn, Na, Sn and Sr.

A very interesting result is the good separation of hazelnuts belonging to the same cultivar (*Romana*), but subjected to different drying processes. This result shows the importance of this step during the shell life of the product, since it can influence the final composition of the hazelnuts. In particular, using D1 (35–40 °C) results in an increase of the concentration of Ca, Mg and Zn and a decrease of the concentration of Mn, with respect to the use of D2 (15–20 °C).

### 3.3. Roasted Hazelnuts

Tables S17–S19 report the composition of roasted and not roasted samples, stored with method B and C at T0 and T4 for O- (S17), RD1- (S18) and RD2- (S19) samples. In regard to roasted hazelnuts, in the case of rO-samples, there is a significant discrepancy between the concentration of K and P found in roasted and non-roasted samples. It is possible to observe that the concentration of K and Mn doubles in the roasted samples, while the opposite behavior is observable for Ba and P. The decrease in the concentration of Ba and P after the roasting process could be explained by a greater affinity of these elements for the oily phase, which is lost during this treatment. The increase of K and Mn concentrations is difficult to explain [45], but the same behavior of K was observed by Adelakun et al. in roasted okra seeds [46]. No differences in the element concentrations were observed among the samples subjected to the different storage methods. This could indicate that the different conservative processes do not have any effect on the subsequent roasting. Obviously, this behavior should be verified by analyzing a higher number of samples. In the case of rR-(D1 and D2)-samples, pre- and post-roasting concentrations are not significantly different (by ANOVA test). The roasting process is a very important step in the shelf life of the hazelnuts, since it enhances and confers the organoleptic properties of the product, but it is also important that the nutritional value of the fresh nuts is not altered during this step. *Romana* hazelnuts seem to maintain their original composition better than the Turkish ones. This behavior seems to be different from that observed for other chemical parameters (proximate composition, fatty acids, total polyphenols, antioxidant activity, and protein fingerprint by SDS-PAGE) investigated in samples of *Tonda Gentile Trilobata* from Italy and from Chile, *Tonda di Giffoni* from Italy, and *Tombul* from Turkey by Locatelli et al. [11]. In their study, no differences were observed depending on the hazelnut origin.

In this study, the loss of lipid content was not considered: roasting slightly increased the oil extractability, but this increment was not significant in laboratory tests. As reported from other researchers [47], the impact of the industrial process would presumably be stronger than that of experimental laboratory roasting adopted in scientific studies, causing a higher damage of membranes and/or higher protein denaturation. In any case, the final concentrations refer to the weight of each sample after roasting to value the variation in the final product.

### 3.4. Pilot Study

The concentrations measured in P-samples collected monthly from the plant during their ripening are reported in Table S20 (hazelnuts sampled in the middle of plantation, Pm) and Table S21 (hazelnuts sampled on the roadside, Pr). No differences can be observed among the trends of the inorganic content obtained for Pm and Pr samples. We applied an ANOVA to our data that shows that the hazelnuts sampled on the roadside in the first month contain a significantly higher concentration of eight elements than those found in Pm samples. This difference was not observed in the hazelnuts sampled during the following months. This behavior reflects the fact that during the first month, the fruit does not possess a shell and is therefore more influenced by the dust composition. In any case, the proximity of the plant to the road does not seem to affect the inorganic composition of the final product. The road near the hazelnut plants is affected only by light traffic, so the input of metals from traffic is low. We conclude that the metals contained in hazelnuts are mainly derived from the soil.

**Figure 4:**
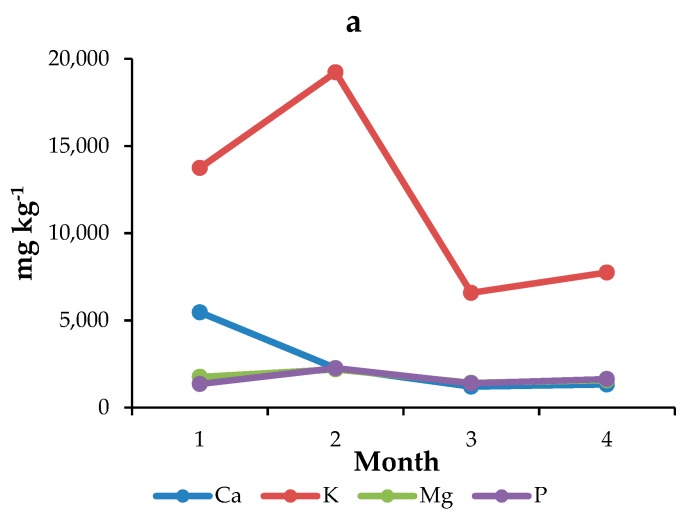

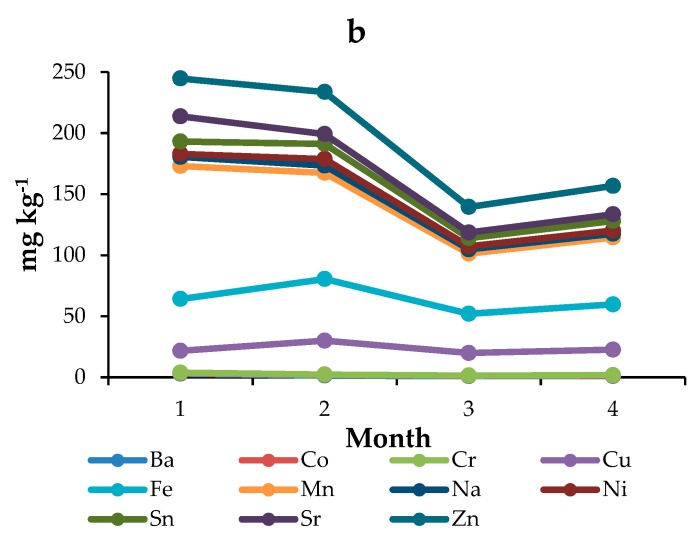
Trends of the element concentrations (mg·kg^−1^) of (**a**) macro-nutrients and (**b**) micro-nutrients during the development of plants in hazelnuts sampled monthly on the roadside.

Figure 4a,b show the trends of macro- and microelements, respectively, for hazelnuts sampled on the roadside. From the first to the second sampling, the concentrations of all the analytes increase. After the second month, it is possible to observe a decrease in element concentrations, probably caused by a loss of water and the formation of the shell; then, the inorganic content remains quite constant. This behavior is in agreement with the results reported by Seyan et al. [48]. The concentrations of Co and Cr remain unvaried throughout all time points.

A comparison among P-samples and the other cultivars is difficult, since the former were analyzed immediately after the harvest, while O- and R-samples were subjected to drying. The element concentrations found in P-samples harvested at four months was comparable with the concentrations of R-samples, since all Piedmont hazelnuts contain a high level of Fe, K, Mn, Na, Sn and Sr. P-samples are also characterized by high levels of Cu and Ni and by a detectable concentration of Co and Cr. These metals (in particular Ni and Co) probably have geogenic origin, since they derive from the composition of the ultramafic soils widespread in Piedmont [49]. Figure S1 shows the biplot obtained by PCA in which four-month P-samples were added to the dataset of the other cultivars. The first and the second PCs retain 49.64% and 22.29% of the total variance, respectively. Obviously, the number of P-samples should be increased to be representative, but from this preliminary treatment, is possible to observe a good separation of not only Italian and Turkish hazelnuts, but also of hazelnuts belonging to different Italian cultivars. Moreover, is possible to see that R-samples are grouped together (D1 and D2), showing that the elemental composition could be a good fingerprint of each cultivar even after industrial treatment. This confirmed the results reported by Locatelli et al. [11] for other analytes; even if the different processes modify the chemical profile of hazelnuts, this preliminary study suggests that the identification of their origin is still possible.

## 4. Conclusions

This study has shown how hazelnuts belonging to different cultivars have a well-defined composition from the point of view of inorganic elements. In particular, the samples belonging to *Romana* cultivars and coming from the Ordu region differ mainly in the concentration of macroelements. The *Romana* hazelnuts are richer in Ca, Fe, K, Mn, Na, Sn, Sr than the Turkish ones, which have higher concentrations of other elements (mainly P and Ba).

Moreover, the treatment and processes involved during the storage and shelf life of the hazelnuts influence the composition of the fruit. In regard to drying processes, it is difficult to assess their specific effect since the three processes (one for O-hazelnuts and two for R-hazelnuts) examined are different and they were applied to hazelnuts belonging to two different cultivars. In any case, the applied temperature and the duration of this step seem to cause a modification of the hazelnut composition during the storage time. Indeed, it is possible to observe that, regardless of the adopted storage method, the *Romana* hazelnuts dried using the D1 method retain their original concentration of K, Mg, Na, P and Sn over time independently of the method adopted. Drying at a lower temperature (D2) in combination with the A-Method seems to be the best, since the hazelnuts appear to better preserve their nutritional power and quality. Unfortunately, after storage, D2 samples show generally higher concentrations of Ni, a catalyst of the self-oxidative process of the hazelnut, which causes rancidity.

Through the chemometric treatment of the data, a good separation between Ordu and *Romana* samples was obtained according to their different inorganic composition. The element concentrations depend on the type of cultivar and the environmental conditions (such as soil, water and temperature). Moreover, it is interesting to observe that *Romana* hazelnuts are grouped on the basis of the drying procedure adopted. No separation was observed among the samples subjected to different storage methods.

In regard to the effect of the roasting process, *Romana* hazelnuts seem to maintain their original composition better than Turkish ones. This behavior seems to be different from that observed for other chemical parameters, for which no differences were observed in samples pre- and post-roasting depending on the hazelnut’s origin.

Regarding the pilot study carried out on Piedmont hazelnuts, it was possible to appreciate a common trend of the concentration of the inorganic component during their growth: most elements increased during the first months of development, then decreased and finally remained approximately constant until complete maturation. Piedmont hazelnuts have a high K content and a composition similar to that of the other Italian cultivars considered in this study. No influence from vehicular traffic was observed, since no differences between the composition of hazelnuts sampled from plants located in the middle of the field and plants located near to the roadside were observed.

In conclusion, the inorganic component detectable with ICP-OES can be considered a good marker for the recognition of the origin and quality of hazelnuts. Moreover, even if the different processes modify the inorganic profile of hazelnuts, this preliminary study suggests that the statistical identification of the cultivar is still possible. The metal content has to be monitored to evaluate loss of nutrients and, on the contrary, possible contamination during all the steps of the shelf life of the product; that is, from the beginning (environment conditions in which the hazelnuts are grown), during the process (to monitor the effect of the different treatments and manipulations), and at the end (in the final product).

Future work will include: (i) the analysis of hazelnuts of the same cultivar grown in different areas to assess the impact of soil composition, the climate, proximity to the sea and various agricultural techniques on the inorganic composition; (ii) the analysis of hazelnuts of different cultivars grown in the same area and sampled in the same year to evaluate the different element absorption capacity of each cultivar; (iii) the study of the effect of roasting step conditions (time, temperature) on the element profile of hazelnuts belonging to different cultivars.

## Figures and Tables

**Table 1:** O-samples and R-samples.

**R-Samples**				
**Drying 1**				
		**R-D1-T0**		
R-D1-AT1	R-D1-BT1	R-D1-CT1	R-D1-DT1	R-D1-ET1
R-D1-AT2	R-D1-BT2	R-D1-CT2	R-D1-DT2	R-D1-ET2
R-D1-AT4	R-D1-BT4	R-D1-CT4	R-D1-DT4	R-D1-ET4
**Drying 2**				
		**R-D2-T0**		
R-D2-AT1	R-D2-BT1	R-D2-CT1	R-D2-DT1	R-D2-ET1
R-D2-AT2	R-D2-BT2	R-D2-CT2	R-D2-DT2	R-D2-ET2
R-D2-AT4	R-D2-BT4	R-D2-CT4	R-D2-DT4	R-D2-ET4
**O-Samples**				
		**O-T0**		
O-AT1	O-BT1	O-CT1	O-DT1	O-ET1
O-AT2	O-BT2	O-CT2	O-DT2	O-ET2
O-AT4	O-BT4	O-CT4	O-DT4	O-ET4

R: *Romana*; O: Ordu; T0, T1, T2, T4: 0, 1, 2, 4 months of storage; A-B-C-D-E: storage methods.

**Table 2:** Samples of roasted hazelnuts.

Roasted Samples	
**rR-D1-T0**	
rR-D1-BT4	rR-D1-CT4
**rR-D2-T0**	
rR-D2-BT4	rR-D2-CT4
**rO-T0**	
rO-BT4	rO-CT4

R: *Romana*; O: Ordu; T0, T1, T2, T4: 0, 1, 2, 4 months of storage; A-B-C-D-E: storage methods; r: roasted.

**Table 3:** Recoveries (%) obtained analyzing Standard Reference Material (SRM) 1573a with different procedures.

Element	Without Internal Standard Correction			With Internal Standard Correction		
	0 mL	1 mL	3 mL	0 mL	1 mL	3 mL
**Al**	89.6	90.8	90	54.4	78.3	80.6
**Ba**	99.3	82.1	84.0	60.2	69.2	75.7
**Ca**	116	115	111	71.7	113	111
**Cd**	108	70.3	82.4	65.5	68.5	74.1
**Co**	122	75.4	79.1	74.1	60.9	71.0
**Cr**	107	68.3	80.8	64.7	65.6	72.6
**Cu**	114	90.8	91.2	112	75.8	82.1
**Fe**	110	86.5	88.4	66.6	72.7	79.6
**K**	116	112	111	90.1	119	119
**Mg**	128	97.7	101	69.8	96.4	99.6
**Mn**	116	90.2	92.3	70.1	75.6	83.1
**Ni**	109	56.2	66.3	65.9	47.3	59.7
**P**	118	99.6	101.9	77.6	84.7	91.7
**Sr**	114	74.8	88.3	70.0	72.8	79.6
**V**	104	56.1	58.4	63.3	49.0	52.0
**Zn**	132	78.6	94.5	80.2	75.8	85.1

## Supplementary Materials

The following are available online: https://www.mdpi.com/1660-4601/17/2/447/s1. Table S1: Concentration of elements (mg·kg^−1^) at T0 for O- and R-samples; Table S2: Concentration of elements (mg·kg^−1^) in O-samples stored with A-Method; Table S3: Concentration of elements (mg·kg^−1^) in O-samples stored with B-Method; Table S4: Concentration of elements (mg·kg^−1^) in O-samples stored with C-Method; Table S5: Concentration of elements (mg·kg^−1^) in O-samples stored with D-Method; Table S6: Concentration of elements (mg·kg^−1^) in O-samples stored with E-Method; Table S7: Concentration of elements (mg·kg^−1^) in R-D1-samples stored with A-Method; Table S8: Concentration of elements (mg·kg^−1^) in R-D1-samples stored with B-Method; Table S9: Concentration of elements (mg·kg^−1^) in R-D1-samples stored with C-Method; Table S10: Concentration of elements (mg·kg^−1^) in R-D1-samples stored with D-Method; Table S11: Concentration of elements (mg·kg^−1^) in R-D1-samples stored with E-Method; Table S12: Concentration of elements (mg·kg^−1^) in R-D2-samples stored with A-Method; Table S13: Concentration of elements (mg·kg^−1^) in R-D2-samples stored with B-Method; Table S14: Concentration of elements (mg·kg^−1^) in R-D2-samples stored with C-Method; Table S15: Concentration of elements (mg·kg^−1^) in R-D2-samples stored with D-Method; Table S16: Concentration of elements (mg·kg^−1^) in R-D2-samples stored with E-Method; Table S17: Concentration of elements (mg·kg^−1^) in O-samples roasted and not roasted, stored with B-Method and C-Method at T0 and T4; Table S18: Concentration of elements (mg·kg^−1^) in R-D1-samples roasted and not roasted, stored with B-Method and C-Method at T0 and T4; Table S19: Concentration of elements (mg·kg^−1^) in R-D2-samples roasted and not roasted, stored with B-Method and C-Method at T0 and T4; Table S20: Concentration of elements (mg·kg^−1^) in Pm samples; Table S21: Concentration of elements (mg·kg^−1^) in Pr samples; Figure S1: Biplot obtained by PCA (PC1 vs. PC2) including hazelnuts from Piedmont in the main dataset.
