# Supplementary material for: The Inorganic Component as a Possible Marker for Quality and for Authentication of the Hazelnut’s Origin
Source: Int J Environ Res Public Health. 2020 Jan 9;17(2):447. doi: 10.3390/ijerph17020447 (PMC7014338; doi:10.3390/ijerph17020447)
Supplement: Supplementary file 1 [file ijerph-17-00447-s001.pdf]

The inorganic component as a possible marker for quality and for authentication of the hazelnut's origin

Paolo Inaudi[1], Agnese Giacomino[1*], Mery Malandrino[2], Carmela La Gioia[2], Eleonora Conca[2], Tanmoy Karak[3] and Ornella Abollino[1]

[1]Department of Drug Science and Technology, University of Torino, 10125 Torino, Italy

[2]Department of Chemistry, University of Torino, 10125 Torino, Italy

[3] Upper Assam Advisory Centre, 786101 Dikom, Assam, India

Supplementary materials

Table S1. Concentration of elements (mg·kg^{-1}) at T0 for O- and R-samples.

Element	Ordu T0	Romana D1 T0	Romana D2 T0
**Ba**	23.4 ± 0.2	2.21 ± 0.15	2.77 ± 0.06
**Ca**	1097 ± 7	1351 ± 27	1200 ± 13
**Cu**	12.3 ± 0.2	13.3 ± 0.8	13.5 ± 0.1
**Fe**	26.1 ± 0.2	38.8 ± 1.1	33.4 ± 0.7
**K**	3472 ± 56	6588 ± 202	6290 ± 57
**Mg**	1342 ± 40	1569 ± 22	1524 ± 9
**Mn**	27.5 ± 1.3	48.6 ± 2.1	60.2 ± 0.1
**Na**	0.06 ± 1 × 10^{-4}	4.91 ± 0.18	4.68 ± 0.27
**Ni**	0.98 ± 0.09	0.68 ± 1 × 10^{-4}	0.62 ± 0.07
**P**	2863 ± 79	1439 ± 21	1435 ± 19
**Sn**	2.77 ± 0.01	6.32 ± 0.86	5.38 ± 0.31
**Sr**	3.95 ± 0.14	23.2 ± 2.1	22.4 ± 0.6
**Zn**	17.2 ± 0.2	18.0 ± 0.2	16.5 ± 0.2
**Sum**	**8865 ± 106**	**11,103 ± 206**	**10,608 ± 62**

Table S2. Concentration of elements (mg·kg^{-1}) in O-samples stored with method A.

Element	T0	T1	T2	T4	Mean
**Ba**	23.4 ± 0.2	44.2 ± 1.5	63.3 ± 1.0	42.5 ± 0.4	50.0 ± 11.6
**Ca**	1097 ± 7	1289 ± 26	1563 ± 10	1423 ± 1	1425 ± 137
**Cu**	12.3 ± 0.2	11.3 ± 0.7	12.9 ± 0.2	14.6 ± 0.2	12.9 ± 1.6
**Fe**	26.1 ± 0.2	26.8 ± 0.1	24.7 ± 0.3	22.3 ± 0.2	24.6 ± 2.3
**K**	3472 ± 56	4007 ± 216	3786 ± 214	3880 ± 122	3891 ± 111
**Mg**	1342 ± 40	1326 ± 2	1496 ± 54	1470 ± 1	1431 ± 91
**Mn**	27.5 ± 1.3	45.8 ± 0.9	43.0 ± 0.3	28.6 ± 1.8	39.1 ± 9.2
**Na**	0.06 ± 1 × 10^{-4}	0.06 ± 1 × 10^{-4}	0.12 ± 1 × 10^{-4}	0.06 ± 1 × 10^{-4}	0.08 ± 0.03
**Ni**	0.98 ± 0.09	1.57 ± 0.02	1.63 ± 0.09	1.23 ± 1 × 10^{-4}	1.48 ± 0.21
**P**	2863 ± 79	2774 ± 60	2885 ± 100	3061 ± 80	2906 ± 145
**Sn**	2.77 ± 0.01	2.78 ± 0.22	3.01 ± 0.05	2.34 ± 0.21	2.71 ± 0.34
**Sr**	3.95 ± 0.14	9.43 ± 0.18	15.8 ± 1.6	16.3 ± 0.4	13.8 ± 3.8
**Zn**	17.2 ± 0.2	16.9 ± 0.1	16.6 ± 0.8	17.3 ± 0.2	16.9 ± 0.3
**Sum**	**8865 ± 106**	**9555 ± 226**	**9854 ± 243**	**9979 ± 146**	

Table S3. Concentration of elements (mg·kg^{-1}) in O-samples stored with method B.

Element	T0	T1	T2	T4	Mean
**Ba**	23.4 ± 0.2	63.6 ± 1.5	50.6 ± 3.3	48.5 ± 2.4	54.2 ± 8.18
**Ca**	1097 ± 7	1316 ± 26	1420 ± 30	1171 ± 9	1302 ± 125
**Cu**	12.3 ± 0.2	11.7 ± 0.7	13.1 ± 0.1	14.8 ± 0.2	13.2 ± 1.53
**Fe**	26.1 ± 0.2	25.4 ± 0.1	27.6 ± 1.1	28.9 ± 0.7	27.3 ± 1.77
**K**	3472 ± 56	3552 ± 216	3279 ± 104	3824 ± 149	3551 ± 272
**Mg**	1342 ± 40	1347 ± 2	1466 ± 32	1507 ± 14	1440 ± 83
**Mn**	27.5 ± 1.3	44.3 ± 0.9	38.6 ± 1.6	30.7 ± 0.4	37.9 ± 6.79
**Na**	0.06 ± 1 × 10^{-4}	0.06 ± 1 × 10^{-4}	0.12 ± 1 × 10^{-4}	0.12 ± 1 × 10^{-4}	0.10 ± 0.03
**Ni**	0.98 ± 0.09	1.10 ± 0.02	1.54 ± 0.07	1.54 ± 0.01	1.39 ± 0.25
**P**	2863 ± 79	2924 ± 60	2860 ± 66	3086 ± 37	2957 ± 116
**Sn**	2.77 ± 0.01	2.83 ± 0.22	3.55 ± 0.15	3.52 ± 0.11	3.30 ± 0.41
**Sr**	3.95 ± 0.14	10.1 ± 0.2	12.4 ± 0.9	14.2 ± 0.8	12.2 ± 2.08
**Zn**	17.2 ± 0.2	15.7 ± 0.1	17.8 ± 0.6	16.4 ± 0.1	16.6 ± 1.10
**Sum**	**8865 ± 106**	**9314 ± 226**	**9190 ± 130**	**9747 ± 154**	

Table S4. Concentration of elements (mg·kg^{-1}) in O-samples stored with method C.

Element	T0	T1	T2	T4	Mean
**Ba**	23.4 ± 0.2	40.7 ± 26.6	29.8 ± 1.6	60.0 ± 0.3	43.5 ± 15.3
**Ca**	1097 ± 7	1274 ± 234	1425 ± 38	1520 ± 84	1406 ± 124
**Cu**	12.3 ± 0.2	13.7 ± 0.7	14.9 ± 0.1	13.5 ± 0.3	14.0 ± 0.73
**Fe**	26.1 ± 0.2	25.7 ± 0.4	27.7± 0.3	26.4 ± 0.4	26.6 ± 0.98
**K**	3472 ± 56	3296 ± 140	4060 ± 162	3932 ± 225	3763 ± 409
**Mg**	1342 ± 40	1390 ± 24	1436 ± 10	1638 ± 41	1488 ± 132
**Mn**	27.5 ± 1.3	31.3 ± 4.8	39.94 ± 1.55	40.41 ± 3.40	37.2 ± 5.10
**Na**	0.06 ± 1 × 10^{-4}	0.06 ± 1 × 10^{-4}	0.12 ± 1 × 10^{-4}	0.12 ± 1 × 10^{-4}	0.10 ± 0.03
**Ni**	0.98 ± 0.09	1.00 ± 0.08	1.22 ± 0.04	1.70 ± 0.39	1.30 ± 0.36
**P**	2863 ± 79	2971 ± 5	3191 ± 9	3179 ± 111	3113 ± 124
**Sn**	2.77 ± 0.01	2.79 ± 0.10	3.88 ± 0.06	3.76 ± 0.85	3.48 ± 0.60
**Sr**	3.95 ± 0.14	7.18 ± 5.05	8.26 ± 0.74	16.44 ± 2.54	10.63 ± 5.06
**Zn**	17.2 ± 0.2	16.9 ± 0.1	17.92 ± 0.04	17.97 ± 0.32	17.61 ± 0.58
**Sum**	**8865 ± 106**	**9070 ± 275**	**10,256 ± 167**	**10,449 ± 268**	

Table S5. Concentration of elements (mg·kg^{-1}) in O-samples stored with method D.

Element	T0	T1	T2	T4	Mean
**Ba**	23.4 ± 0.2	22.7 ± 0.02	58.9 ± 1.5	42.6 ± 3.1	41.4 ± 18.1
**Ca**	1097 ± 7	1397 ± 3	1260 ± 13	1504 ± 87	1387 ± 122
**Cu**	12.3 ± 0.2	13.0 ± 0.7	13.9 ± 0.3	14.6 ± 1 × 10^{-4}	13.8 ± 0.81
**Fe**	26.1 ± 0.2	25.0 ± 1.0	26.7 ± 0.8	28.6 ± 0.7	26.8 ± 1.80
**K**	3472 ± 56	3731 ± 48	3355 ± 42	3942 ± 610	3676 ± 297
**Mg**	1342 ± 40	1470 ± 44	1454 ± 31	1485 ± 48	1470 ± 15
**Mn**	27.5 ± 1.3	25.9 ± 0.6	38.5 ± 0.5	38.1 ± 0.8	34.2 ± 7.18
**Na**	0.06 ± 1 × 10^{-4}	0.12 ± 1 × 10^{-4}	0.12 ± 1 × 10^{-4}	0.12 ± 1 × 10^{-4}	0.12 ± 1 × 10^{-4}
**Ni**	0.98 ± 0.09	0.85 ± 0.09	1.19 ± 0.01	1.42 ± 0.08	1.15 ± 0.29
**P**	2863 ± 79	2965 ± 216	2844 ± 46	3029 ± 93	2946 ± 94
**Sn**	2.77 ± 0.01	2.10 ± 1.40	3.18 ± 0.05	3.77 ± 0.06	3.02 ± 0.85
**Sr**	3.95 ± 0.14	19.05 ± 1.25	11.7 ± 0.7	6.97 ± 0.19	12.6 ± 6.09
**Zn**	17.2 ± 0.2	16.9 ± 0.4	16.01 ± 0.02	17.7 ± 1.2	16.8 ± 0.84
**Sum**	**8865 ± 106**	**9689 ± 226**	**9083 ± 71**	**10,114 ± 625**	

Table S6. Concentration of elements (mg·kg^{-1}) in O-samples stored with method E.

Element	T0	T1	T2	T4	Mean
**Ba**	23.4 ± 0.2	57.1 ± 0.8	48.8 ± 0.6	47.3 ± 1.8	51.1 ± 5.28
**Ca**	1097 ± 7	1485 ± 19	1342 ± 24	1307 ± 22	1378 ± 94
**Cu**	12.3 ± 0.2	15.0 ± 0.1	13.4 ± 0.1	13.2 ± 0.1	13.9 ± 1.03
**Fe**	26.1 ± 0.2	29.7 ± 1.1	26.8 ± 0.7	26.11 ± 0.93	27.5 ± 1.88
**K**	3472 ± 56	3603 ± 158	3331 ± 30	3528 ± 99	3487 ± 140
**Mg**	1342 ± 40	1428 ± 14	1427 ± 7	1353 ± 29	1403 ± 43
**Mn**	27.5 ± 1.3	28.8 ± 0.2	35.6 ± 1.6	40.0 ± 2.4	34.8 ± 5.63
**Na**	0.06 ± 1 × 10^{-4}	0.12 ± 1 × 10^{-4}	0.09 ± 0.04	0.12 ± 1 × 10^{-4}	0.11 ± 0.02
**Ni**	0.98 ± 0.09	0.99 ± 0.04	1.10 ± 0.04	1.29 ± 0.13	1.13 ± 0.15
**P**	2863 ± 79	3026 ± 77	3035 ± 14	2775 ± 22	2945 ± 148
**Sn**	2.77 ± 0.01	4.23 ± 0.16	3.21 ± 0.17	2.79 ± 0.73	3.41 ± 0.74
**Sr**	3.95 ± 0.14	15.1 ± 0.4	8.36 ± 0.06	10.6± 0.6	11.4 ± 3.42
**Zn**	17.2 ± 0.2	17.3 ± 0.1	16.7 ± 0.5	17.6 ± 0.5	17.2 ± 0.47
**Sum**	**8865 ± 106**	**9710 ± 177**	**9289 ± 42**	**9,122 ± 108**	

Table S7. Concentration of elements (mg·kg^{-1}) in R-D1-samples stored with method A.

Element	T0	T1	T2	T4	Mean
**Ba**	2.21 ± 0.15	1.60 ± 0.13	1.64 ± 0.01	1.88 ± 0.05	1.71 ± 0.15
**Ca**	1351 ± 27	1300 ± 4	1426 ± 62	1208 ± 5	1311 ± 110
**Cu**	13.3 ± 0.8	14.1 ± 1.3	13.6 ± 0.7	14.9 ± 0.11	14.2 ± 0.65
**Fe**	38.8 ± 1.1	35.3 ± 1.5	35.1 ± 3.1	37.9 ± 0.6	36.1 ± 1.54
**K**	6588 ± 202	6447 ± 1404	5914 ± 270	7424 ± 251	6595 ± 766
**Mg**	1569 ± 22	1617 ± 136	1636 ± 7	1626 ± 18	1626 ± 9
**Mn**	48.6 ± 2.1	37.3 ± 2.2	35.4 ± 0.9	64.15 ± 0.94	45.6 ± 16.1
**Na**	4.91 ± 0.18	3.89 ± 0.38	4.28 ± 0.59	6.42 ± 0.28	4.87 ± 1.36
**Ni**	0.68 ± 1 × 10^{-4}	0.66 ± 0.20	0.77 ± 1 × 10^{-4}	0.77 ± 0.04	0.74 ± 0.07
**P**	1439 ± 21	1666 ± 167	1627 ± 31	1502 ± 27	1599 ± 86
**Sn**	6.32 ± 0.86	6.72 ± 0.15	7.20 ± 0.52	5.73 ± 0.58	6.55 ± 0.75
**Sr**	23.2 ± 2.1	16.7 ± 0.6	20.5 ± 0.7	39.3 ± 3.5	25.5 ± 12.09
**Zn**	18.0 ± 0.2	17.2 ± 0.9	17.0 ± 1.3	17.5 ± 0.1	17.24 ± 0.24
**Sum**	**11,103 ± 206**	**11,163 ± 1420**	**10,738 ± 279**	**11,949 ± 253**	

Table S8. Concentration of elements (mg·kg^{-1}) in R-D1-samples stored with method B.

Element	T0	T1	T2	T4	Mean
**Ba**	2.21 ± 0.15	1.52 ± 0.05	1.25 ± 0.10	1.45 ± 0.06	1.41 ± 0.14
**Ca**	1351 ± 27	1320 ± 72	1147 ± 62	1349 ± 46	1272 ± 109
**Cu**	13.3 ± 0.8	13.8 ± 0.9	14.5 ± 1.1	12.6 ± 0.1	13.6 ± 0.97
**Fe**	38.8 ± 1.1	41.9 ± 0.8	38.5 ± 5.3	41.3 ± 0.8	40.5 ± 1.82
**K**	6588 ± 202	6035 ± 132	6057 ± 435	6601 ± 226	6231 ± 321
**Mg**	1569 ± 22	1614 ± 40	1550 ± 32	1652 ± 2	1605 ± 51
**Mn**	48.6 ± 2.1	47.5 ± 0.1	52.8 ± 3.5	37.4 ± 0.1	45.9 ± 7.85
**Na**	4.91 ± 0.18	6.29 ± 0.88	5.45 ± 1.31	6.69 ± 0.05	6.14 ± 0.63
**Ni**	0.68 ± 1 × 10^{-4}	1.08 ± 0.33	1.00 ± 0.09	0.74 ± 0.08	0.94 ± 0.18
**P**	1439 ± 21	1623 ± 57	1434 ± 51	1429 ± 34	1492 ± 114
**Sn**	6.32 ± 0.86	8.09 ± 0.07	7.39 ± 0.45	6.53 ± 0.33	7.34 ± 0.79
**Sr**	23.2 ± 2.1	37.3 ± 1.6	17.5 ± 2.2	20.7 ± 0.3	25.2 ± 10.6
**Zn**	18.0 ± 0.2	19.1 ± 0.4	17.3 ± 1.9	18.9 ± 0.4	18.4 ± 0.94
**Sum**	**11,103 ± 206**	**10,769 ± 166**	**10,344 ± 444**	**11,177 ± 233**	

Table S9. Concentration of elements (mg·kg⁻¹) in R-D1-samples stored with method C.

Element	T0	T1	T2	T4	Mean
**Ba**	2.21 ± 0.15	1.52 ± 0.05	1.41 ± 0.10	2.16 ± 0.06	1.70 ± 0.41
**Ca**	1351 ± 27	1525 ± 72	1251 ± 61	1221 ± 4	1332 ± 167
**Cu**	13.3 ± 0.8	15.1 ± 0.9	13.6 ± 1.1	12.4 ± 0.2	13.7 ± 1.34
**Fe**	38.8 ± 1.1	39.7 ± 0.8	36.6 ± 5.3	38.7 ± 0.4	38.4 ± 1.60
**K**	6588 ± 202	6383 ± 132	5761 ± 435	6257 ± 32	6134 ± 328
**Mg**	1569 ± 22	1600 ± 40	1540 ± 32	1601 ± 1	1581 ± 35
**Mn**	48.6 ± 2.1	33.1 ± 0.1	60.5 ± 3.5	40.9 ± 1.7	44.8 ± 14.1
**Na**	4.91 ± 0.18	5.11 ± 0.88	4.46 ± 1.31	6.41 ± 1.10	5.33 ± 0.99
**Ni**	0.68 ± 1 × 10^{-4}	0.94 ± 0.33	0.72 ± 0.09	0.63 ± 0.08	0.76 ± 0.16
**P**	1439 ± 21	1520 ± 57	1564 ± 51	1377 ± 23	1487 ± 98
**Sn**	6.32 ± 0.86	6.79 ± 0.07	7.55 ± 0.45	6.00 ± 0.60	6.78 ± 0.78
**Sr**	23.2 ± 2.1	20.8 ± 1.6	25.3 ± 2.2	35.8 ± 0.6	27.3 ± 7.70
**Zn**	18.0 ± 0.2	18.2 ± 0.3	16.7 ± 1.9	18.5 ± 0.2	17.80 ± 0.97
**Sum**	**11,103 ± 206**	**11,169 ± 166**	**10,283 ± 443**	**10,618 ± 40**	

Table S10. Concentration of elements (mg·kg⁻¹) in R-D1-samples stored with method D.

Element	T0	T1	T2	T4	Mean
**Ba**	2.21 ± 0.15	1.78 ± 0.04	2.61 ± 0.06	1.69 ± 0.09	2.02 ± 0.51
**Ca**	1351 ± 27	1283 ± 16	1267 ± 38	1059 ± 47	1203 ± 125
**Cu**	13.3 ± 0.8	15.0 ± 0.1	13.2 ± 0.2	12.2 ± 0.2	13.5 ± 1.42
**Fe**	38.8 ± 1.1	37.5 ± 0.4	37.03 ± 0.70	33.5 ± 1.5	36.01 ± 2.21
**K**	6588 ± 202	6778 ± 74	6650 ± 560	6230 ± 63	6553 ± 287
**Mg**	1569 ± 22	1702 ± 48	1547 ± 92	1434 ± 47	1561 ± 134
**Mn**	48.6 ± 2.1	53.2 ± 0.1	40.6 ± 0.4	41.6 ± 1.2	45.1 ± 7.01
**Na**	4.91 ± 0.18	6.01 ± 1.11	4.93 ± 0.14	6.88 ± 0.04	5.94 ± 0.98
**Ni**	0.68 ± 1 × 10^{-4}	1.25 ± 1 × 10^{-4}	0.66 ± 0.04	0.48 ± 0.04	0.80 ± 0.41
**P**	1439 ± 21	1525 ± 52	1415 ± 115	1343 ± 16	1428 ± 92
**Sn**	6.32 ± 0.86	6.65 ± 0.06	7.03 ± 0.17	5.30 ± 0.52	6.32 ± 0.91
**Sr**	23.2 ± 2.1	21.8 ± 0.1	36.3 ± 0.7	19.8 ± 0.7	26.0 ± 8.99
**Zn**	18.0 ± 0.2	17.4 ± 0.1	17.8 ± 0.1	15.7 ± 0.7	16.98 ± 1.09
**Sum**	**11,103 ± 206**	**11,449 ± 104**	**11,039 ± 580**	**10,203 ± 93**	

Table S11. Concentration of elements (mg·kg⁻¹) in R-D1-samples stored with method E.

Element	T0	T1	T2	T4	Mean
**Ba**	2.21 ± 0.15	1.71 ± 0.10	1.83 ± 0.10	1.79 ± 0.07	1.78 ± 0.06
**Ca**	1351 ± 27	1169 ± 36	1210 ± 37	1160 ± 16	1180 ± 27
**Cu**	13.3 ± 0.8	12.5 ± 0.4	11.7 ± 0.3	14.4 ± 0.4	12.9 ± 1.38
**Fe**	38.8 ± 1.1	38.8 ± 1.1	35.6 ± 1.0	35.2 ± 1.1	36.6 ± 1.97
**K**	6588 ± 202	6097 ± 205	6735 ± 60	6054 ± 869	6289 ± 386
**Mg**	1569 ± 22	1544 ± 92	1605 ± 1	1541 ± 129	1563 ± 36
**Mn**	48.6 ± 2.1	33.0 ± 1.8	48.8 ± 1.8	39.7 ± 1.7	40.5 ± 7.96
**Na**	4.91 ± 0.18	7.06 ± 0.50	5.78 ± 0.75	5.35 ± 0.07	6.07 ± 0.89
**Ni**	0.68 ± 1 × 10^{-4}	0.66 ± 0.09	0.45 ± 1 × 10^{-4}	0.62 ± 0.07	0.58 ± 0.11
**P**	1439 ± 21	1507 ± 91	1362 ± 9	1432 ± 136	1434 ± 72
**Sn**	6.32 ± 0.86	5.58 ± 0.12	5.45 ± 0.14	5.14 ± 0.52	5.39 ± 0.23
**Sr**	23.2 ± 2.1	26.9 ± 0.3	30.8 ± 2.0	19.3 ± 0.7	25.7 ± 5.87
**Zn**	18.0 ± 0.2	18.7 ± 0.8	16.2 ± 0.9	16.8 ± 0.4	17.2 ± 1.32
**Sum**	**11,103 ± 206**	**10,462 ± 245**	**11,069 ± 72**	**10,325 ± 889**	

Table S12. Concentration of elements (mg·kg^{-1}) in R-D2-samples stored with method A.

Element	T0	T1	T2	T4	Mean
**Ba**	2.77 ± 0.06	1.94 ± 0.02	1.67 ± 0.10	2.26 ± 0.06	1.96 ± 0.29
**Ca**	1200 ± 13	1066 ± 3	1107 ± 37	1176 ± 23	1117 ± 55
**Cu**	13.5 ± 0.1	14.1 ± 0.2	14.5 ± 0.3	14.1 ± 0.1	14.2 ± 0.23
**Fe**	33.4 ± 0.7	32.4 ± 0.2	31.8 ± 1.0	34.1 ± 1.2	32.8 ± 1.16
**K**	6290 ± 57	6367 ± 144	5735 ± 60	6334 ± 570	6145 ± 355
**Mg**	1524 ± 9	1544 ± 7	1408 ± 1	1510 ± 29	1,487 ± 71
**Mn**	60.2 ± 0.1	57.8 ± 1.3	70.3 ± 1.8	65.0 ± 0.1	64.4 ± 6.25
**Na**	4.68 ± 0.27	5.61 ± 0.78	4.64 ± 0.75	5.67 ± 0.50	5.30 ± 0.58
**Ni**	0.62 ± 0.07	0.85 ± 0.05	0.77 ± 0.01	1.12 ± 0.01	0.91 ± 0.18
**P**	1435 ± 19	1548 ± 5	1375 ± 9	1395 ± 30	1439 ± 94
**Sn**	5.38 ± 0.31	5.45 ± 0.35	5.03 ± 0.14	5.43 ± 1.24	5.30 ± 0.24
**Sr**	22.4 ± 0.6	14.1 ± 0.1	14.4 ± 2.0	17.0 ± 0.1	15.2 ± 1.56
**Zn**	16.5 ± 0.2	17.3 ± 1.0	17.4 ± 0.9	17.8 ± 0.9	17.5 ± 0.29
**Sum**	**10,608 ± 62**	**10,675 ± 144**	**9785 ± 71**	**10,577 ± 572**	

Table S13. Concentration of elements (mg·kg^{-1}) in R-D2-samples stored with method B.

Element	T0	T1	T2	T4	Mean
**Ba**	2.77 ± 0.06	1.69 ± 0.02	2.38 ± 0.04	1.79 ± 0.31	1.95 ± 0.37
**Ca**	1200 ± 13	1076 ± 3	1018 ± 31	1047 ± 22	1047 ± 29
**Cu**	13.5 ± 0.1	15.3 ± 0.2	13.2 ± 0.2	13.2 ± 0.8	13.9 ± 1.20
**Fe**	33.4 ± 0.7	34.2 ± 0.2	29.5 ± 1.0	31.3 ± 1.1	31.6 ± 2.37
**K**	6290 ± 57	5958 ± 144	6232 ± 104	5918 ± 644	6036 ± 171
**Mg**	1524 ± 9	1,514 ± 7	1,405 ± 21	1,467 ± 1	1,462 ± 55
**Mn**	60.2 ± 0.1	59.7 ± 1.3	55.9 ± 0.3	60.9 ± 5.6	58.8 ± 2.63
**Na**	4.68 ± 0.27	4.09 ± 0.78	6.93 ± 2.00	6.09 ± 1.30	5.70 ± 1.46
**Ni**	0.62 ± 0.07	0.83 ± 0.05	0.78 ± 0.08	0.81 ± 0.05	0.81 ± 0.02
**P**	1435 ± 19	1697 ± 5	1257 ± 38	1349 ± 89	1434 ± 232
**Sn**	5.38 ± 0.31	5.99 ± 0.35	4.80 ± 1.23	4.20 ± 0.05	5.00 ± 0.91
**Sr**	22.4 ± 0.6	16.3 ± 0.1	16.9 ± 0.3	16.5 ± 2.9	16.6 ± 0.28
**Zn**	16.5 ± 0.2	15.7 ± 1.0	16.5 ± 0.2	16.1 ± 0.8	16.1 ± 0.41
**Sum**	**10,608 ± 62**	**10,400 ± 144**	**10,059 ± 117**	**9932 ± 651**	

Table S14. Concentration of elements (mg·kg^{-1}) in R-D2-samples stored with method C.

Element	T0	T1	T2	T4	Mean
**Ba**	2.77 ± 0.06	1.81 ± 0.06	1.99 ± 0.05	1.69 ± 0.27	1.83 ± 0.15
**Ca**	1200 ± 13	1073 ± 62	1108 ± 11	1038 ± 3	1073 ± 35
**Cu**	13.5 ± 0.1	14.6 ± 0.4	15.8 ± 0.1	13.1 ± 1.0	14.5 ± 1.38
**Fe**	33.4 ± 0.7	34.2 ± 1.1	31.6 ± 0.3	32.0 ± 1.7	32.6 ± 1.37
**K**	6290 ± 57	6095 ± 112	5690 ± 8	4997 ± 674	5594 ± 555
**Mg**	1524 ± 9	1492 ± 3	1487 ± 24	1394 ± 55	1458 ± 56
**Mn**	60.2 ± 0.1	54.2 ± 0.7	59.2 ± 1.4	61.0 ± 5.7	58.1 ± 3.50
**Na**	4.68 ± 0.27	6.11 ± 1.03	6.33 ± 1.52	5.35 ± 0.41	5.93 ± 0.51
**Ni**	0.62 ± 0.07	0.89 ± 0.01	0.93 ± 0.20	0.94 ± 0.19	0.92 ± 0.03
**P**	1435 ± 19	1471 ± 9	1396 ± 28	1329 ± 137	1399 ± 71
**Sn**	5.38 ± 0.31	6.06 ± 0.25	4.97 ± 0.66	5.57 ± 0.39	5.54 ± 0.55
**Sr**	22.4 ± 0.6	16.0 ± 0.1	13.9 ± 0.2	15.6 ± 3.7	15.1 ± 1.11
**Zn**	16.5 ± 0.2	16.1 ± 0.1	18.0 ± 0.8	16.1 ± 0.4	16.7 ± 1.10
**Sum**	**10,608 ± 62**	**10,281 ± 128**	**9834 ± 39**	**8909 ± 690**	

Table S15. Concentration of elements (mg·kg^{-1}) in R-D2-samples stored with method D.

Element	T0	T1	T2	T4	Mean
**Ba**	2.77 ± 0.06	1.62 ± 0.04	1.53 ± 0.05	2.21 ± 0.14	1.79 ± 0.37
**Ca**	1200 ± 13	1172 ± 123	1059 ± 11	1149 ± 12	1127 ± 60
**Cu**	13.5 ± 0.1	18.2 ± 0.15	16.3 ± 0.12	13.8 ± 0.6	16.1 ± 2.21
**Fe**	33.4 ± 0.7	36.6 ± 2.2	32.4 ± 0.33	29.8 ± 1.1	32.9 ± 3.47
**K**	6290 ± 57	6675 ± 141	6590 ± 8	5509 ± 595	6258 ± 650
**Mg**	1524 ± 9	1533 ± 43	1556 ± 24	1521 ± 25	1537 ± 18
**Mn**	60.2 ± 0.1	57.8 ± 0.7	55.4 ± 1.4	59.9 ± 1.9	57.7 ± 2.21
**Na**	4.68 ± 0.27	9.16 ± 5.48	5.19 ± 1.52	7.04 ± 0.80	7.13 ± 1.98
**Ni**	0.62 ± 0.07	1.14 ± 0.27	0.92 ± 0.20	0.63 ± 0.07	0.90 ± 0.26
**P**	1435 ± 19	1610 ± 29	1426 ± 28	1264 ± 12	1433 ± 173
**Sn**	5.38 ± 0.31	7.67 ± 0.63	5.81 ± 0.66	3.76 ± 0.14	5.75 ± 1.96
**Sr**	22.4 ± 0.6	14.7 ± 0.5	13.5 ± 0.2	18.0 ± 0.8	15.4 ± 2.31
**Zn**	16.5 ± 0.2	16.6 ± 1.8	16.7 ± 0.8	16.0 ± 0.2	16.4 ± 0.37
**Sum**	**10,608 ± 62**	**11,153 ± 194**	**10,779 ± 39**	**9594 ± 596**	

Table S16. Concentration of elements (mg·kg^{-1}) in R-D2-samples stored with method E.

Element	T0	T1	T2	T4	Mean
**Ba**	2.77 ± 0.06	2.22 ± 0.04	1.92 ± 0.02	2.08 ± 0.13	2.07 ± 0.15
**Ca**	1200 ± 13	1350 ± 123	1083 ± 29	1094 ± 18	1176 ± 151
**Cu**	13.5 ± 0.1	14.7 ± 0.1	16.8 ± 0.3	13.6 ± 0.7	15.0 ± 1.59
**Fe**	33.4 ± 0.7	36.5 ± 2.2	32.5 ± 1*10^{-4}	34.1 ± 1.2	34.4 ± 2.02
**K**	6290 ± 57	6309 ± 141	6122 ± 78	6139 ± 183	6190 ± 103
**Mg**	1524 ± 9	1674 ± 43	1537 ± 2	1532 ± 46	1581 ± 81
**Mn**	60.2 ± 0.1	74.2 ± 0.7	58.0 ± 1.4	63.4 ± 1.4	65.2 ± 8.26
**Na**	4.68 ± 0.27	3.74 ± 5.48	6.03 ± 0.37	6.00 ± 0.11	5.26 ± 1.31
**Ni**	0.62 ± 0.07	0.89 ± 0.27	0.69 ± 0.01	1.02 ± 0.01	0.87 ± 0.17
**P**	1435 ± 19	1576 ± 29	1505 ± 33	1444 ± 32	1508 ± 66
**Sn**	5.38 ± 0.31	7.07 ± 0.63	4.31 ± 0.21	5.47 ± 0.62	5.62 ± 1.39
**Sr**	22.4 ± 0.6	22.4 ± 0.5	15.8 ± 0.9	14.8 ± 0.7	17.7 ± 4.11
**Zn**	16.5 ± 0.2	17.9 ± 1.8	17.3 ± 0.2	17.3 ± 0.7	17.5 ± 0.35
**Sum**	**10,608 ± 62**	**11,089 ± 194**	**10,400 ± 90**	**10,368 ± 192**	

Table S17. Concentration of elements (mg·kg^{-1}) in O-samples roasted and not roasted, stored with method B and C at T0 and T4.

Element	T0	T0 r	T4 B	T4 B r	T4 C	T4 C r
**Ba**	23.4 ± 0.2	0.58 ± 0.04	48.5 ± 2.4	0.75 ± 0.05	60.0 ± 0.3	0.53 ± 0.04
**Ca**	1097 ± 7	1558 ± 33	1171 ± 9	1442 ± 27	1520 ± 84	1483 ± 16
**Cu**	12.3 ± 0.2	14.7 ± 0.2	14.8 ± 0.2	14.4 ± 0.2	13.5 ± 0.3	14.7 ± 0.4
**Fe**	26.1 ± 0.2	30.8 ± 0.6	28.9 ± 0.7	27.6 ± 0.1	26.4 ± 0.4	24.8 ± 0.7
**K**	3472 ± 56	6763 ± 610	3824 ± 149	5772 ± 290	3932 ± 225	6049 ± 504
**Mg**	1342 ± 40	1509 ± 8	1507 ± 14	1520 ± 59	1638 ± 41	1462 ± 11
**Mn**	27.5 ± 1.3	59.1 ± 0.8	30.7 ± 0.4	52.5 ± 3.5	40.41 ± 3.40	55.9 ± 4.6
**Na**	0.06 ± 1 × 10^{-4}	3.53 ± 0.26	0.12 ± 1 × 10^{-4}	2.59 ± 0.02	0.12 ± 1 × 10^{-4}	3.10 ± 0.03
**Ni**	0.98 ± 0.09	1.40 ± 0.03	1.54 ± 0.01	1.65 ± 0.01	1.70 ± 0.39	1.55 ± 0.08
**P**	2863 ± 79	1604 ± 28	3086 ± 37	1576 ± 49	3179 ± 111	1604 ± 23
**Sn**	2.77 ± 0.01	6.38 ± 0.11	3.52 ± 0.11	5.15 ± 0.29	3.76 ± 0.85	5.24 ± 1.13
**Sr**	3.95 ± 0.14	5.79 ± 0.25	14.2 ± 0.8	13.8 ± 0.3	16.44 ± 2.54	8.32 ± 0.38
**Zn**	17.2 ± 0.2	19.3 ± 0.7	16.4 ± 0.1	18.5 ± 0.4	17.97 ± 0.32	20.2 ± 0.4
**Sum**	**8865 ± 106**	**11,576 ± 612**	**9747 ± 154**	**10,447 ± 301**	**10,449 ± 268**	**10,733 ± 505**

Table S18. Concentration of elements ($mg \cdot kg^{-1}$) in R-D1-samples roasted and not roasted, stored with method B and C at T0 and T4.

Element	T0	T0 r	T4 B	T4 B r	T4 C	T4 C r
**Ba**	2.21 ± 0.15	1.42 ± 0.01	1.45 ± 0.06	1.26 ± 0.07	2.16 ± 0.06	1.22 ± 0.05
**Ca**	1351 ± 27	1344 ± 45	1349 ± 46	1183 ± 98	1221 ± 4	1246 ± 15
**Cu**	13.3 ± 0.8	16.0 ± 0.1	12.6 ± 0.1	14.9 ± 0.2	12.4 ± 0.2	11.6 ± 0.3
**Fe**	38.8 ± 1.1	35.9 ± 0.6	41.3 ± 0.8	36.2 ± 2.1	38.7 ± 0.4	36.8 ± 0.5
**K**	6588 ± 202	7669 ± 836	6601 ± 226	7069 ± 194	6257 ± 32	6489 ± 123
**Mg**	1569 ± 22	1644 ± 44	1652 ± 2	1611 ± 83	1601 ± 1	1530 ± 10
**Mn**	48.6 ± 2.1	55.6 ± 5.0	37.4 ± 0.1	32.5 ± 1.2	40.9 ± 1.7	48.7 ± 3.4
**Na**	4.91 ± 0.18	4.96 ± 0.11	6.69 ± 0.05	5.52 ± 0.04	6.41 ± 1.10	5.23 ± 0.34
**Ni**	$0.68 \pm 1 \times 10^{-4}$	0.89 ± 0.06	0.74 ± 0.08	0.62 ± 0.04	0.63 ± 0.08	0.72 ± 0.12
**P**	1439 ± 21	1630 ± 63	1429 ± 34	1385 ± 97	1377 ± 23	1350 ± 53
**Sn**	6.32 ± 0.86	8.20 ± 0.59	6.53 ± 0.33	8.31 ± 0.77	6.00 ± 0.60	9.48 ± 1.43
**Sr**	23.2 ± 2.1	27.3 ± 0.8	20.7 ± 0.3	23.1 ± 1.5	35.8 ± 0.6	28.0 ± 0.5
**Zn**	18.0 ± 0.2	20.4 ± 0.3	18.9 ± 0.4	17.9 ± 0.6	18.5 ± 0.2	17.6 ± 0.4
**Sum**	**11,103 ± 206**	**12,458 ± 841**	**11,177 ± 233**	**11,388 ± 252**	**10,618 ± 40**	**10,775 ± 135**

Table S19. Concentration of elements ($mg \cdot kg^{-1}$) in R-D2-samples roasted and not roasted, stored with method B and C at T0 and T4.

Element	T0	T0 r	T4 B	T4 B r	T4 C	T4 C r
**Ba**	2.77 ± 0.06	1.68 ± 0.04	1.79 ± 0.31	2.30 ± 0.03	1.69 ± 0.27	1.74 ± 0.29
**Ca**	1200 ± 13	1056 ± 15	1047 ± 22	1174 ± 8	1038 ± 3	1136 ± 73
**Cu**	13.5 ± 0.1	15.9 ± 0.6	13.2 ± 0.8	16.7 ± 0.5	13.1 ± 1.0	18.3 ± 0.1
**Fe**	33.4 ± 0.7	33.2 ± 1.1	31.3 ± 1.1	33.6 ± 0.4	32.0 ± 1.7	31.4 ± 0.8
**K**	6290 ± 57	6158 ± 57	5918 ± 644	7486 ± 455	4997 ± 674	6909 ± 629
**Mg**	1524 ± 9	1587 ± 42	1467 ± 1	1700 ± 13	1394 ± 55	1658 ± 44
**Mn**	60.2 ± 0.1	57.7 ± 1.9	60.9 ± 5.6	58.7 ± 1.1	61.0 ± 5.7	63.7 ± 0.5
**Na**	4.68 ± 0.27	4.67 ± 0.57	6.09 ± 1.30	5.05 ± 0.51	5.35 ± 0.41	4.75 ± 0.55
**Ni**	0.62 ± 0.07	1.19 ± 0.01	0.81 ± 0.05	0.90 ± 0.04	0.94 ± 0.19	0.78 ± 0.04
**P**	1435 ± 19	1539 ± 5	1349 ± 89	1570 ± 4	1329 ± 137	1548 ± 59
**Sn**	5.38 ± 0.31	7.66 ± 0.18	4.20 ± 0.05	7.91 ± 0.14	5.57 ± 0.39	7.79 ± 0.47
**Sr**	22.4 ± 0.6	18.5 ± 0.1	16.5 ± 2.9	19.1 ± 0.2	15.6 ± 3.7	16.5 ± 2.4
**Zn**	16.5 ± 0.2	20.0 ± 0.6	16.1 ± 0.8	18.4 ± 0.7	16.1 ± 0.4	16.5 ± 0.5
**Sum**	**10,608 ± 62**	**10,500 ± 73**	**9932 ± 651**	**12,094 ± 455**	**8909 ± 690**	**11,413 ± 637**

Table S20. Concentration of elements ($mg \cdot kg^{-1}$) in Pm-samples.

Element	1m	2m	3m	4m
**Ba**	2.33 ± 0.10	1.50 ± 0.04	0.85 ± 0.01	0.95 ± 0.05
**Ca**	5196 ± 3	3825 ± 64	1562 ± 45	1907 ± 8
**Co**	0.49 ± 0.01	0.72 ± 0.01	0.51 ± 0.04	0.48 ± 0.01
**Cr**	0.43 ± 0.10	0.24 ± 0.01	0.12 ± 0.01	0.18 ± 0.01
**Cu**	14.1 ± 0.3	26.0 ± 1.0	17.7 ± 1.1	17.3 ± 0.5
**Fe**	34.5 ± 3.9	54.0 ± 0.8	30.5 ± 0.9	32.8 ± 0.2
**K**	10,196 ± 74	21,818 ± 23	8402 ± 658	6743 ± 402
**Mg**	1716 ± 63	2832 ± 58	1551 ± 60	1529 ± 65
**Mn**	69.7 ± 2.2	94.0 ± 3.5	50.3 ± 0.4	56.6 ± 1.1
**Na**	9.07 ± 0.31	7.66 ± 0.05	4.36 ± 0.74	4.03 ± 1.46
**Ni**	2.07 ± 0.11	4.92 ± 0.10	2.17 ± 0.05	2.03 ± 0.02
**P**	1161 ± 24	2819 ± 70	1624 ± 64	1477 ± 21

**Sn**	7.23 ± 0.89	12.5 ± 0.3	5.65 ± 0.20	7.39 ± 2.17
**Sr**	19.8 ± 0.9	12.8 ± 0.4	5.88 ± 0.06	7.24 ± 0.33
**Zn**	28.5 ± 0.4	43.9 ± 0.3	23.9 ± 1.5	24.2 ± 0.2
**Sum**	**18,457 ± 100**	**28,720 ± 114**	**13,281 ± 665**	**11,809 ± 408**

Table S21. Concentration of elements (mg·kg^{-1}) in Pr-samples.

Element	1m	2m	3m	4m
**Ba**	2.96 ± 0.11	1.41 ± 0.01	0.91 ± 0.01	1.03 ± 0.02
**Ca**	5463 ± 38	2263 ± 31	1204 ± 23	1328 ± 3
**Co**	0.36 ± 0.01	0.60 ± 0.01	0.30 ± 0.01	0.36 ± 0.01
**Cr**	0.45 ± 0.04	0.21 ± 0.04	0.18 ± 0.01	0.38 ± 0.3
**Cu**	17.9 ± 0.3	27.8 ± 1.3	18.5 ± 0.7	20.9 ± 0.3
**Fe**	42.5 ± 0.3	50.5 ± 0.4	32.1 ± 0.6	37.1 ± 0.8
**K**	13,745 ± 181	19,227 ± 381	6581 ± 380	7746 ± 243
**Mg**	1760 ± 15	2192 ± 118	1424 ± 51	1568 ± 27
**Mn**	109 ± 1	87.0 ± 2.6	49.3 ± 0.9	54.9 ± 0.6
**Na**	7.48 ± 0.23	5.93 ± 0.86	3.48 ± 0.16	3.00 ± 0.38
**Ni**	2.49 ± 0.11	5.39 ± 0.02	2.41 ± 0.04	2.61 ± 0.04
**P**	1357 ± 34	2260 ± 92	1401 ± 79	1636 ± 14
**Sn**	10.1 ± 0.5	12.4 ± 0.9	6.78 ± 0.17	7.87 ± 0.05
**Sr**	20.6 ± 1.0	8.00 ± 0.15	4.57 ± 0.13	5.42 ± 0.22
**Zn**	31.0 ± 0.7	34.5 ± 0.1	20.9 ± 0.3	23.3 ± 0.2
**Sum**	**22,543 ± 189**	**26,176 ± 411**	**10,749± 392**	**10,799 ± 245**

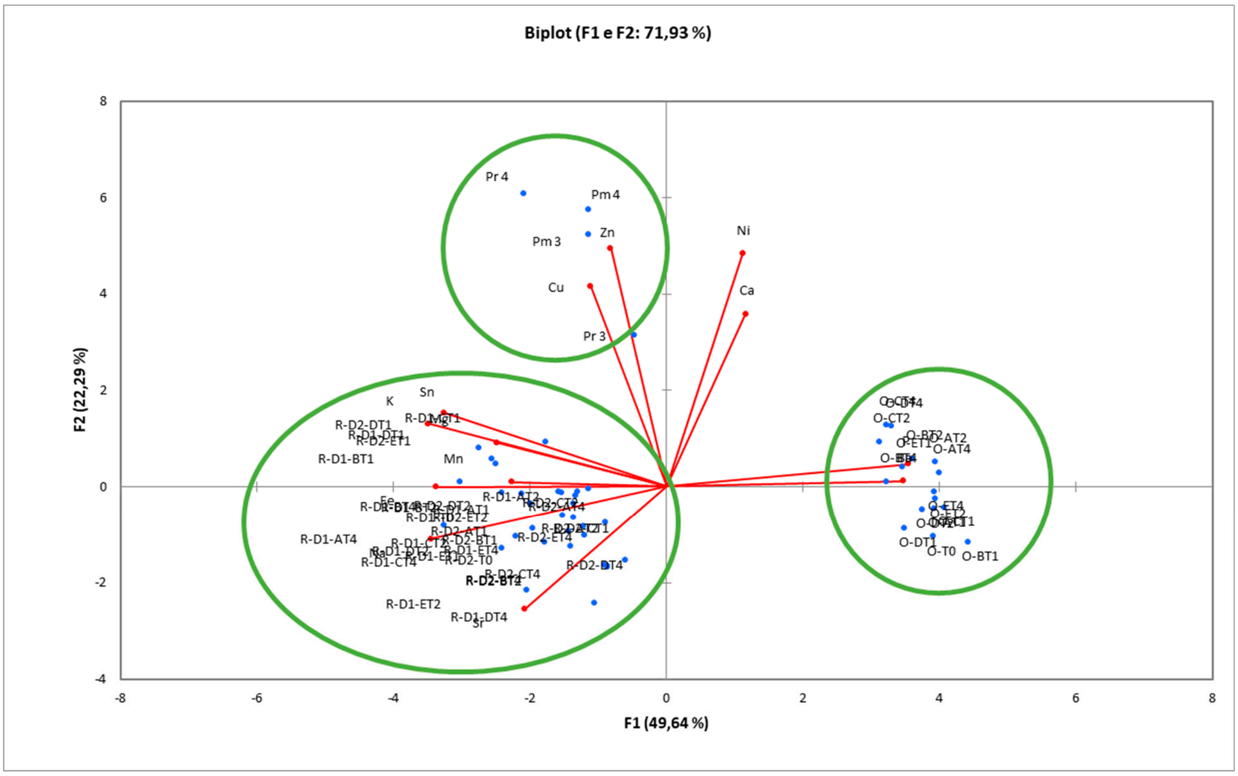

Figure S1. Biplot obtained by PCA (PC1 vs. PC2) including hazelnuts from Piedmont in the main dataset.
